# An Overview of Central Board for the Accreditation of Healthcare Institutions in Saudi Arabia: A Narrative Review

**DOI:** 10.7759/cureus.95137

**Published:** 2025-10-22

**Authors:** Majed Alturbag, Albatol Alyahya

**Affiliations:** 1 Radiology, Eye Care Center, Buraydah, SAU; 2 Eye Care, Eye Care Center, Buraydah, SAU

**Keywords:** cbahi, compliance barriers, healthcare accreditation, infection control, patient safety, quality of care, saudi arabia

## Abstract

This study aimed to synthesise current Saudi-based literature on (i) factors that enable or hinder compliance with Central Board for Accreditation of Healthcare Institutions (CBAHI) requirements and (ii) the recognised impact of accreditation on patient safety and infection control. A narrative review was undertaken. Nineteen empirical studies published between January 2015 and May 2025 were retrieved. Study characteristics, methodological designs, and principal outcomes were extracted, and findings were thematically grouped into enablers, infection control outcomes, patient safety effects, pre-/post-quality comparisons, and barriers to compliance. Most investigations were hospital-based cross-sectional surveys or audits conducted in Riyadh, Makkah/Jeddah, and Dammam; four involved primary healthcare centres and two examined mixed hospital networks. Successful implementation of CBAHI was consistently linked to strong executive leadership, continuous staff training, robust quality management systems, and adequate resource allocation. Infection control standards (especially committee functionality and isolation protocols) and hand-hygiene adherence presented marked improvements after accreditation, with private and larger hospitals outperforming public and smaller facilities. Five of six patient safety studies reported significant gains in incident reporting culture, medication error reduction, or healthcare-associated infection rates, yet changes in hard clinical outcomes and occupational safety climate were less consistent. Key barriers to sustained compliance included weak IT infrastructure, shortages of personal protective equipment (PPE), time pressure, high staff turnover, inadequate management support, and an underdeveloped national health information system. CBAHI accreditation is associated with measurable advances in infection control and selected patient safety indicators across Saudi healthcare settings, but its full potential is inhibited by organisational and systemic bottlenecks. Embedding accreditation within everyday practice, investing in digital infrastructure and workforce development, and strengthening leadership accountability are important to transforming CBAHI from an episodic certification exercise into a continuous driver of healthcare excellence.

## Introduction and background

Compliance with healthcare accreditation standards is essential for institutions seeking to ensure patient safety and deliver high-quality care [1]. Accreditation serves as a cornerstone for continuous improvement and institutional accountability [2]. Nonetheless, many healthcare facilities struggle with full compliance due to multifaceted challenges, including systemic, organisational, and human factors [3].

The Central Board for Accreditation of Healthcare Institutions (CBAHI) is mandatory in Saudi Arabia. National policy requires all public and private healthcare delivery facilities to comply with CBAI's standards and obtain accreditation through its survey process; the Essential Safety Requirements (ESR) represent the minimum, mandatory patient safety standards. Established in 2005, CBAHI’s mission is to define, promote, and enforce evidence-based quality standards throughout the Kingdom’s healthcare system [4]. For hospitals, CBAHI accreditation has been explicitly linked to operating license renewal, and mandatory accreditation has been extended program by program to other facility types [5]. The framework evaluates healthcare facilities against predefined criteria grouped into three primary domains: structural, procedural and outcome-based standards [6,7]. Beyond accreditation, CBAHI provides professional education, training, consultation services, and comprehensive feedback to guide continuous quality improvement [8].

CBHAI’s strategic goals extend beyond domestic reform, aiming for international recognition, development of tailored healthcare programs, and the training of qualified accreditation surveyors [9,10]. Currently, CBAHI oversees seven national programs, including those for hospitals, primary healthcare centres, dental clinics, clinical laboratories and blood banks, ambulatory care, home healthcare, and acute coronary syndrome services [9]. As of 2018, over 75% of hospitals had successfully met CBAHI’s ESR, marking a substantial increase in nationwide participation and commitment to quality standards [10,11].

Despite growing acceptance of accreditation as a driver of healthcare reform, compliance remains hindered by barriers such as inadequate staff training, limited resources, and infrastructural constraints [12-14]. These challenges can negatively impact care delivery and patient outcomes [15]. While international literature affirms the positive role of accreditation in improving healthcare quality, there remains a need to examine the specific factors influencing compliance in the Saudi context [15].

This narrative review addresses that gap by synthesising evidence on the implementation of CBAHI standards in Saudi Arabia. It explores healthcare professionals’ perceptions, identifies systemic and institutional barriers, and evaluates the impact of accreditation on healthcare quality. In doing so, the review offers a more nuanced understanding of how accreditation can be effectively implemented and sustained across diverse healthcare settings in alignment with national health reform goals.

## Review

Methods

To build the evidence base for this narrative review, a structured literature search was undertaken. The databases PubMed, Embase, CINAHL, Scopus, Web of Science, and the Saudi Digital Library and the official websites of the Central Board were searched for Accreditation of Healthcare Institutions and the Saudi Patient Safety Center, covering January 1, 2015, to May 31, 2025. Search terms had included variants of the CBAHI and ESR, Saudi Arabia, patient safety, infection control, accreditation, and compliance. Studies had been included if they were empirical, conducted in Saudi Arabia in hospital or primary care settings, evaluated Central Board accreditation or ESR, and reported compliance and or infection control or patient safety outcomes in English or Arabic. Studies had been excluded if they were non-empirical, outside Saudi Arabia, unrelated to Central Board standards, conference abstracts without extractable data, duplicates, or reports lacking compliance or safety outcomes. Titles, abstracts, and full texts had been screened independently by two reviewers, with discrepancies resolved by discussion, and data had been extracted on setting, design, sample, measures, and main results.

Study Overview

Nineteen primary studies met the eligibility criteria and formed the body of evidence for this review. Publication dates spanned almost a decade, from January 2015 to May 2025, reflecting the progressive roll-out of the CBAHI and other local quality-improvement initiatives across the Kingdom. Geographical coverage was broad: the central (Riyadh) and western (Makkah/Jeddah) regions contributed the largest share of studies, followed by the eastern (Dammam/Al-Ahsa), southern (Aseer), and northern provinces. Most investigations were hospital-based, but four evaluated primary healthcare centres and two examined mixed networks of public and private facilities. The concentration of studies in Riyadh and Makkah/Jeddah likely reflects their higher density of tertiary hospitals and academic centres and earlier adoption of Central Board activities, whereas the southern and northern regions have smaller populations and fewer research-active facilities.

Study Designs 

Methodologically, the body of evidence is dominated by cross-sectional survey or audit designs (n = 14), typically employing structured questionnaires, retrospective chart reviews or point-prevalence observational checklists. Three studies employed mixed-methods approaches, combining quantitative findings with focus-group or key-informant interviews to explore underlying attitudes and organisational dynamics. A single retrospective cohort applied machine learning (random forest) analytics to predict documentation compliance at scale, and one quasi-experimental before-and-after study assessed the effectiveness of accreditation-driven infection control training.

Enablers of CBAHI Implementation

The successful implementation of healthcare accreditation programs such as the CBAHI in Saudi Arabia requires a confluence of organisational, human, and systemic enablers. ‘Enablers’ are defined as facility-level factors that support compliance with Central Board standards; programme-level context is reported only where it frames local implementation. Two empirical studies provide valuable insights into the facilitators that drive the effective execution of CBAHI standards within the Saudi healthcare landscape (Table 1).

**Table 1:** Enabling factors for accreditation programs

Authors	Sample size	Methodology	Location	Facility type	Enablers identified	Summary outcome
Althumairi et al. (2022) [8]	437 hospitals	Quantitative cross-sectional	20 regions, Saudi Arabia	Public and private Hospitals	Enabler: private hospitals outperformed the public consistently.	Private hospitals showed significantly better Essential Safety Requirements (ESR) compliance (84% vs. 68%). Larger hospitals performed better on some metrics.
Alsaedi et al. (2023) [16]	22 professionals	Qualitative (thematic analysis)	Madinah, Saudi Arabia	Ministry of Health (MOH) hospitals	Enablers included leadership commitment, trained staff, enhanced quality culture, resource availability, and simplified standards.	Identified four core themes (human capital, quality systems, resources, Central Board for Accreditation of Healthcare Institutions (CBAHI)-specific solutions) for successful CBAHI execution.

Alsaedi et al. (2023) conducted a qualitative study in five Ministry of Health (MOH) hospitals in Madinah, Saudi Arabia, and identified four key thematic areas enabling successful implementation: human capital development, quality management enhancement, resources availability, and CBAHI-specific strategic solutions [16]. Leadership commitment and staff engagement were emphasised as foundational, with effective CBAHI implementation relying heavily on trained and motivated personnel. Qualitative management systems, including the promotion of a hospital-wide quality culture and adherence to standardised protocols, were also instrumental in developing consistent performance. Notably, the availability of both medical and non-medical resources was cited as essential for maintaining the momentum beyond the accreditation cycle. Moreover, participants highlighted the need for simplified standards, targeted training and voluntary participation to cultivate intrinsic motivation rather than mere compliance. This study highlights the importance of contextualising accreditation programs within hospital realities and integrating accreditation into daily routines rather than treating it as an episodic exercise.

In a complementary nationwide quantitative study, Althumairi et al. (2022) assessed the compliance with the CBAHI’s ESR across 437 hospitals in Saudi Arabia between 2016 and 2018 [8]. The study found that hospital type and size influence compliance levels. Private hospitals demonstrated higher average compliance (84%) compared to public hospitals (68%), and larger hospitals outperformed smaller ones. This suggests that institutional capacity, financial autonomy, and operational flexibility are significant enablers. Furthermore, compliance rates improved over time, possibly reflecting growing institutional familiarity with accreditation processes and internal quality culture maturation. Notably, the study emphasised the critical role of ESR standards in improving patient safety, serving as a stepping stone toward broader accreditation goals. The findings support sustained monitoring, capacity building, and a tailored approach to accreditation rollout across diverse hospital settings. These studies illustrate that successful implementation of the CBAHI program is not solely dependent on regulatory mandates but rather hinges on the interplay of leadership, staff improvement, infrastructure, and adaptive accreditation strategies. Embedding accreditation within organisational culture and aligning it with intrinsic quality goals can transform it from a checklist-driven burden to a genuine driver of healthcare excellence.

Infection Prevention and Control

Infection prevention and control (IPC) is a foundation of patient safety and quality assurance in healthcare delivery. The CBAHI in Saudi Arabia has integrated IPC standards as a key component of its ESR, aiming to unify practices across public and private healthcare settings. The integration of such standards has been associated with improved outcomes, as evidenced by recent studies evaluating the real-world impact of CBAHI-driven IPC protocols. Two studies were identified and synthesised in the context of this narrative review to assess the impact of CBAHI standards on infection control practices across healthcare settings in Saudi Arabia (Table 2).

**Table 2:** Infection prevention and control ANOVA: analysis of variance, KSA: Kingdom of Saudi Arabia, MOH: Ministry of Health, PHC: primary healthcare, ESR: Essential Safety Requirements, IPC: infection prevention and control, PPE: personal protective equipment

Authors	Sample size	Methodology	Location	Facility type	Main factors	Summary outcome	r
Abduljawad (2022) [17]	440 hospitals	Quantitative (ANOVA, correlation)	Nationwide, KSA	Public and private hospitals	ESR improved IPC; private hospitals performed better; weak link with bed size.	Accreditation improved IPC scores over three years; scores rose in both IPC 4 and IPC 15 standards.	r = 0.19–0.24 (bed size vs. IPC)
Alameer et al. (2018) [18]	320 participants, 16 PHCs	Descriptive cross-sectional	Makkah, KSA	MOH PHC dental clinics	Training, PPE, and good satisfaction; barriers include a lack of incentives and staff shortage.	High compliance with IPC measures and satisfaction in 16 PHCs.	Not reported

A nationwide quantitative study by Abduljawad (2023) investigated the effect of CBAHI ESR implementation on infection control quality across 440 hospitals in Saudi Arabia between 2016 and 2018 [17]. The study focused on two critical IPC standards: IPC4 (oversight committee functionality) and IPC15 (isolation facilities and protocols). Results revealed significant improvements in IPC scores over a three-year span, especially in private hospitals. For IPC4, which encompasses oversight functions, scores improved from 78% in 2016 to 94% in 2018. Similarly, IPC15 scores, relating to structural and procedural isolation measures, increased from 66% to 70%, with private institutions outperforming public ones. Statistical analysis using mixed ANOVA confirmed the significance of these trends (P < 0.001). Interestingly, the correlation between hospital bed size and IPC performance was weak as reported by Abduljawad (2022), suggesting that standard implementation was effective irrespective of institutional scale.

Complementing these findings, Alameer et al. (2018) assessed IPC adherence in a more localised setting, i.e., dental clinics within 16 primary healthcare centres in Makkah [18]. Through a structured questionnaire distributed among dental practitioners, technicians, and community members (n = 320), the study evaluated satisfaction, personal protective equipment (PPE) usage, sterilisation protocols, and awareness of infection control practices. The majority of participants reported full compliance with IPC standards, particularly in the availability and usage of personal protective equipment and sterilisation procedures. However, challenges such as the absence of hazard pay or other financial incentives for high-risk roles, together with staffing shortages, were cited as barriers to optimal infection control compliance. Despite these limitations, the study concluded that the CBAHI’s implementation positively impacted infection control perceptions and practices at the primary care level.

These studies highlight a progressive trajectory in infection control across both hospital and primary care settings in Saudi Arabia. While improvements were more marked in private institutions, overall compliance with IPC standards has increased since the enforcement of the CBAHI’s ESR in 2016. These findings highlight the value of structured accreditation in elevating the baseline of infection control nationwide. Moreover, the result suggests a need for continued investment in training, staffing, and equitable policy implementation to sustain and scale these gains across the healthcare system.

Patient Safety Outcomes

A consistent thread across the Saudi literature is that accreditation by the CBAHI is tightly coupled with measurable shifts in patient-safety culture and outcomes. Across the Saudi literature, only five primary investigations met the a priori inclusion criteria (original, quantitative data on CBAHI implementation and any patient safety or care quality endpoint) (Table 3).

**Table 3:** Patient safety HC: healthcare, MOH: Ministry of Health, CBAHI: Central Board for Accreditation of Healthcare Institutions, FMS: Facility Management and Safety, ANOVA: analysis of variance, KPI: Key Performance Indicator, IOM: Institute of Medicine, QI: quality improvement, LASA: look-alike/sound-alike (medications)

Authors	Sample size / scope	Methodology	Location	Facility type	Key barriers / enablers identified	Summary outcome	Domain	r
Abdurabuh et al. (2024) [19]	340 HC workers	Cross-sectional survey (CBAHI-FMS); descriptive stats + ANOVA/t-test + multiple regression	Makkah Region, KSA	5 public MOH hospitals	Enablers: disaster training, facility safety, security	Accreditation is linked to better patient-safety culture; leadership and QI are weak.	Patient-safety culture	0.82
Pappiya et al. (2022) [20]	110 nurses	Descriptive cross-sectional questionnaire	Najran Region, KSA	Secondary MOH hospital	Enablers: ↑ accountability, med-process, IPC. Barriers: little change in LASA meds and basic precautions	Nurses report improvements in several safety areas, some unchanged.	Nursing / patient-safety	-
Alsaedi et al. (2023) [21]	5 MOH hospitals (60-mo data series)	Retrospective 60-month segmented regression (12 mo pre, 36 during, 12 post)	Madinah Region, KSA	General MOH hospitals	Enablers: sustained ↓ incident reports, med-errors, nosocomial infections	Significant progressive improvement in IOM “safety” indicators	IOM safety	-
Salami & Bhatti (2023) [22]	102 hospital employees	Online cross-sectional survey; multiple regression (R² = 0.79)	Al-Ahsa, KSA	Large general hospital	Enablers: compliance with hazardous materials, medical equipment, fire safety, and security standards. Barriers: facility-safety, utilities, and emergency-prep standards are not predictive; external-disaster drills are weak.	FMS compliance explains 79% of the variance in perceived quality; certain programs drive quality gains.	FMS / healthcare quality	0.79
Alqarni et al. (2021) [23]	3 hospitals; 15 mo pre / 36 mo post	Retrospective cohort; Mann–Whitney U comparisons of efficiency and safety KPIs	Makkah, KSA	2 MOH tertiary + 1 MOI tertiary	Barrier: accreditation did not change most efficiency or safety metrics; only bed-turnover differed after reaccreditation.	No significant impact of accreditation or reaccreditation on efficiency or patient-safety measures	Efficiency and patient safety	-

Four studies reported statistically significant improvements in at least one safety or quality metric after accreditation, one study demonstrated mixed or programme-specific gains, and one failed to show any measurable benefit. Multidisciplinary safety-culture work by Abdurabuh et al. (2024) surveyed 340 front-line staff in the five public hospitals and found higher composite safety-culture scores for accredited facilities, with gender, age and nationality as significant covariates [19]. Leadership/commitment emerged as the weakest domain, signalling an implementation gap that persists after accreditation. A nurse-focused cross-sectional survey in Najran (n = 110) by Pappiya et al. (2022) showed that 42.7% of respondents “strongly agreed” that CBAHI standards improved professional accountability and incident reporting; parallel gains were seen for medication safety and documentation processes [20]. Using segmented (piece-wise) time-series regression across 60 months of routine data, Alsaedi et al. (2023) documented sustained, step-wise reductions in incident reports, medication errors and nosocomial infection rates throughout pre-, during-, and post-accreditation phases in five MOH hospitals in Madinah [21]. Furthermore, in an organisation-level compliance analysis (n = 102 staff responses), Salami and Bhatti (2023) correlated self-reported adherence to the seven Facility Management and Safety (FMS) standards with perceived service quality (R2 = 0.79) [22]. Only hazardous materials, medical equipment, fire safety, and security sub-domains independently predicted quality scores, emphasising the need to tailor improvement programmes to the most influential standards. Moreover, a retrospective cohort study in three Makkah hospitals by Alqarni et al. (2021) compared 15 months pre- vs. 36 months post-accreditation and found no significant change in efficiency (bed occupancy, ALOS) or core safety indicators, except for a modest increase in bed-turnover rate; the authors called for more rigorous impact designs [23]. Five patient safety indicators showed non-significant worsening, while only one of five quality improvement measures (blood component wastage) improved, highlighting that accreditation alone may not guarantee laboratory-sector gains [24]. Across studies, the clearest improvements were in incident reporting culture, medication safety processes such as look-alike sound-alike segregation and double checks, and infection prevention and control adherence, including personal protective equipment use and isolation protocols. Effects on hard clinical outcomes and on occupational safety climate were mixed or not significant.

Collectively, these six studies affirm that CBAHI-accredited facilities tend to report modest and domain-specific improvements, with several outcomes mixed or not significant across settings, patient safety cultures, and, in many occurrences, better safety outcomes. Similarly, regional evidence from Dubai demonstrated that accreditation surveys positively influenced healthcare professionals’ perceptions of medication safety practices and safety culture, reinforcing the broader role of accreditation in advancing patient safety across healthcare systems [25]. Heterogeneity in design and follow-up periods tempers causal certainty, but the weight of evidence aligns CBAHI standards, predominantly FMS, medication management and incident-reporting requirements, with tangible safety benefits across diverse Saudi settings.

Quality of Care Before and After Accreditation

A total of six empirical investigations were found that compared dimensions of service quality before versus after a facility or a group of facilities that had obtained certification from the CBAHI (Table 4). Taken together, these studies illuminate how heterogeneous the reported effects are across settings, clinical foci, and measurement strategies, but they also provide a useful composite picture of what accreditation has and has not delivered so far in the Saudi context.

**Table 4:** Assess the quality of care before and after CBAHI CBAHI: Central Board for Accreditation of Healthcare Institutions, PHCC: primary healthcare center, PHC: primary healthcare, HCW: healthcare worker, SCQ: Safety Culture Questionnaire, KSA: Kingdom of Saudi Arabia, PCAT: Primary Care Assessment Tool, CARF: Commission on Accreditation of Rehabilitation Facilities, CBAHI: Central Board for Accreditation of Healthcare Institutions, JCI: Joint Commission International

Authors (year)	Sample	Setting	Design and tool(s)	What was compared	Main findings (before ⇄ after the CBAHI)	Summary outcome
Al-Muraikhi et al. (2020) [26]	15 PHCCs (5 accredited, 10 non-accredited)	Eastern Province, KSA	Analytic cross-sectional; PCAT director interview	Mean PCAT score and domains (first contact, continuity, coordination, etc.); p-values	Total PCAT: CBAHI = 287 vs. non-CBAHI = 247 ( +40; p = 0.100 ns). Largest gap in community orientation (p = 0.083).	CBAHI sites trend better, but differences are not yet significant → questions about real-world impact and the need for larger, national evaluation.
Al-Khaldi et al. (2020) [27]	1 PHCC, 429 diabetic records (2018)	Abha	Retrospective audit of structures, processes, and outcomes against the CBAHI chronic-disease chapter	Compliance scores (0–3) for 10 structure and 10 process items; metabolic / risk-factor control	Structures mostly ‘fully met’, but health education, referral, and lab only ‘partially met’. Process gaps in annual labs/eye exam (≈30% missed). Good control: DM 28%, HTN 71%, lipids 54%.	Applying the CBAHI checklist pinpointed weak links (education, labs, and referral). Even with high structural scores, clinical outcomes (DM control) remain low, underscoring the need to push beyond paperwork to clinical practice.
Alameer et al. (2020) [28]	5 newly accredited PHCs	250 service-users (25 ♂ + 25 ♀ / centre), Makkah	Descriptive cross-sectional; patient questionnaire (satisfaction, complaints, proposals)	Satisfaction with nine service areas (clinic, nursing, lab, etc.) pre-perception vs. post-accreditation perception	72-96% of respondents ‘satisfied’ with clinic and nursing care; very low reported dissatisfaction. Participants linked improvements in the work environment and error reduction to CBAHI certification.	Early user feedback suggests accreditation boosted perceived service quality and safety culture in Makkah PHCs, although the study lacked a pre-accreditation baseline.
Alahmadi et al. (2020) (29)	24 PHCCs, 322 HCWs (162 accredited, 160 non-accredited)	Medina	Comparative cross-sectional; self-administered SP questionnaire	Knowledge and compliance with infection-control standard precautions	Knowledge: no sig. difference overall; higher in accredited only for environmental cleaning and waste disposal. Practice: good compliance 99.4% (accred.) vs. 86.3% (non-accred.), p < 0.001	CBAHI accreditation drives day-to-day compliance with IPC measures, but upgrades in HCW knowledge are modest – ongoing training must accompany accreditation.
Al Shami (2025) (30)	47 peer-reviewed studies (2000-2024) covering physiotherapy services in	KSA	Structured narrative review (CARF, CBAHI, JCI, Planetree frameworks)	Patient outcomes, staff development, and service standardisation pre/post accreditation	Across frameworks, accreditation linked to ↓ clinical errors, ↑ guideline adherence, enhanced staff competency; CBAHI-accredited units showed better functional recovery and satisfaction than non-accredited peers in included analytic studies.	For rehab/physio, accreditation (incl. CBAHI) is a strategic lever for safer, more consistent, patient-centred care—policy support and sustained integration recommended.
Alibraheem & Zytoon (2020) (31)	6 MOH hospitals (3 accredited, 3 non-accredited); 450 valid SCQ responses		Cross-sectional; Safety Culture Questionnaire (25 items, 6 dimensions)	Mean scores across safety-culture dimensions	Non-accredited hospitals scored higher in most dimensions (management commitment, training, communication, support, work env.); only risk-appreciation higher in accredited. Differences sig. for management commitment.	Current CBAHI standards do not translate into a stronger safety culture for staff; occupational-safety requirements should be strengthened in future CBAHI revisions.

The earliest of the six studies focused on primary care accessibility and continuity in 43 MOH centres in Dammam and Khobar. Using the Primary Care Assessment Tool (PCAT), Al-Muraikhi and colleagues (2020) showed that CBAHI-accredited clinics returned a higher mean total score (287 vs. 247) than non-accredited clinics, yet the difference was not statistically significant, and the largest gap was confined to the “community-orientation” domain [26]. The authors concluded that the accreditation badge, by itself, had not translated into demonstrably superior first-contact access or coordination of care, an observation that immediately raised questions about the validity of the standard for day-to-day primary care work.

A more granular audit was carried out at the Al-Manhal family practice centre in Abha, where electronic charts for 429 patients with diabetes were reviewed for a full CBAHI cycle [27]. Structural inputs (staffing, availability of instruments, and drug supply) met most standards, but only 28% of patients achieved good glycaemic control after accreditation was in place. Laboratory completeness and eye examination rates remained only partially compliant, mirroring earlier pre-accreditation audits. In other words, clinical process indicators improved modestly, yet patient-level outcomes barely moved, underscoring that accreditation can highlight gaps but will not close them without supplementary disease management investment. Furthermore, Alameer and co-workers (2020) adopted a patient-experience lens in five newly accredited PHCs in Makkah [28]. Surveying 250 male and female attendees, they documented large majorities expressing satisfaction with overall services, with no respondents in three of the five centres declaring outright dissatisfaction. While the authors attributed this favourable pattern to the recent CBAHI survey, the absence of pre-accreditation baseline data and the wide variation in satisfaction across service departments (e.g., X-ray vs. clinic reception) suggested that part of the improvement may have stemmed from centre-specific initiatives rather than the accreditation process alone. Moving from ambulatory care to infection control practice, Alahmadi’s (2020) comparative cross-sectional study enrolled 322 healthcare workers in Medina [29]. Although knowledge scores about standard precautions did not differ between accredited and non-accredited clusters, self-reported compliance was dramatically higher in CBAHI sites (99.4% ‘good practice’ vs 86.3%). Multivariable analysis linked accreditation to significantly better use of personal protective equipment, environmental cleaning and waste disposal behaviours, implying that the CBAHI infection control chapter can exert a measurable, behaviour-level effect even when cognitive knowledge has plateaued.

A broader rehabilitation perspective was provided by Al Shami’s (2025) narrative synthesis of accreditation frameworks (Commission on Accreditation of Rehabilitation Facilities (CARF), Planetree, Joint Commission International (JCI), and Central Board for Accreditation of Healthcare Institutions (CBAHI)) on physiotherapy outcomes across Saudi hospitals [30]. While the review aggregated multiple data sources, the Saudi-specific segment highlighted that CBAHI-accredited therapy departments reported higher functional independence gains and tighter adherence to standardised treatment protocols than their non-accredited counterparts. Nevertheless, the authors cautioned that robust ‘before/after’ data remained scarce and that many published improvements were drawn from single-centre case reports rather than controlled comparisons, highlighting a need for prospective outcome registries. Finally, Alibraheen and Zytoon (2020) evaluated occupational safety culture in six Jazan Hospitals [31]. Contrary to expectations, mean scores for management commitment, safety communication, and training were lower in accredited than in non-accredited hospitals, with only the personal priority dimension scoring slightly higher post-accreditation. The authors suggested that the CBAHI’s hospital manual, while rigorous on patient safety and clinical governance items, pays too little attention to staff safety, an omission that could explain why accreditation failed to engender a stronger safety climate.

Across the six studies, two consistent themes emerge. First accreditation does tend to standardise structure and raise staff compliance with prescriptive procedures, most clearly in infection control and, to a lesser extent, in chronic disease documentation. Second, hard clinical outcomes and broader organisational culture are much slower to change and, in some domains (notably occupational safety), may even stagnate or regress unless CBAHI standards are explicitly revised or supplemented. Methodological limitations, cross-sectional designs, single-site analysis, self-reported data, and absence of long pre-accreditation baselines clearly moderate these findings, yet the corpus as a whole suggested that the promised quality dividends of accreditation are real but contingent on parallel quality improvement work, leadership engagement, and indicator-level feedback loops rather than on the certification status alone.

Barriers to Compliance With CBAHI Program Standards

Recent evidence pinpoints a multilayered set of obstacles that keep Saudi healthcare organisations from meeting CBAHI requirements. Three empirical and review papers by Kabrah et al. (2024), Alkhurayji et al. (2025), and the earlier sector overview by Almasabi (2013) offer complementary perspectives on where, why, and how non-compliance arises (Table 5) [32-34].

**Table 5:** Factors for non-compliance HCP: healthcare professional, PHC: primary healthcare, MOH: Ministry of Health, PPE: personal protective equipment

Study	Sample size	Research design	Region	Type of facility	Key factors for non-compliance
Almasabi (2013) (4)		Narrative review	Kingdom-wide	Public and private hospitals	Public-sector-dominated financing model, shortage and high turnover of qualified workforce, and limited senior management commitment
Alkhurayji et al. (2025) (33)	10 primary studies (5 in meta-analyses)	Systematic review with random-effects meta-correlation	Kingdom-wide (multi-site)	Mixed: hospitals and PHCs	Insufficient or irregular training, lack of PPE/material resources, ownership (public vs. private), bed-capacity constraints
Kabrah et al. (2024) (32)	364 HCPs	Cross-sectional survey	20 government hospitals across Saudi Arabia	Government (MOH) hospitals	Time pressure, inefficient IT tools, weak communication, limited admin/top-leadership support, perceived irrelevance to daily duties

Kabrah and colleagues surveyed 364 professionals in 20 government hospitals already accredited by the CBAHI and found that, although overall attitude towards the standards was positive, day-to-day implementation faltered for practical reasons [32]. The most frequently endorsed barriers were inadequate information technology tools (59.6% agreement), chronic time constraints associated with high clinical workload (58.8%), and perceptions of weak top management support (58.2%). Poor horizontal communication across wards and departments was also cited by more than half the respondents. Significantly, very few staff questioned the intrinsic value of accreditation; instead, they felt hamstrung by organisational infrastructure that failed to translate paper standards into workable routines.

The national systematic review and meta-analysis by Alkhurayji et al. (2025) pooled data from 10 primary studies, five were used for meta-analysis to quantify determinants of non-compliance [33]. While the aggregate correlation between any single factor and adherence was modest (r ~0.26), several variables consistently predicted lower compliance scores: irregular or insufficient staff training, shortages of PPE, large public-sector bed capacity, and absence of structured implementation strategies. Conversely, facilities that embedded continuous training programmes reported better compliance, underlining the importance of capacity building rather than one-off orientation sessions.

Almasabi’s (2013) broader narrative review situates these hospital-level findings within systemic bottlenecks that have hindered Saudi quality initiatives for decades [34]. He argues that the predominantly public financial model dilutes accountability for meeting eternal benchmarks, while the heavy reliance on expatriate clinicians with turnover rates approaching 37% undermines continuity of quality improvement efforts. Furthermore, an underdeveloped national health information infrastructure and shortages of qualified local quality assurance personnel impede timely monitoring and feedback, both prerequisites for sustained compliance.

These three studies converge on a trio of difficulties: 1) resource and infrastructure gaps, notably IT systems, essential equipment and protected staff time; 2) organisational-behavioural barriers such as limited leadership commitment, weak intra-organisational communication and high workforce turnover; and 3) macrosystem constraints, including financial structures and shortages of trained quality professionals. Addressing CBAHI non-compliance, therefore, requires an integrated strategy that couples on-the-ground capacity-building (robust IT, recurrent training, adequate supplies) with stronger leadership accountability and national-level investment in health-information systems and workforce development.

Discussion

To strengthen the evidence base surrounding the impact of CBAHI accreditation, future research should aim to systematically evaluate its association with key patient safety indicators, morbidity, and mortality rates. While preliminary findings suggest that accreditation may contribute to enhanced clinical outcomes, current evidence remains limited by methodological constraints, such as small sample sizes, cross-sectional designs, and insufficient control of confounding variables [16,30]. There is a critical need for large-scale, multi-centre, and longitudinal studies that assess patient outcomes both before and after accreditation implementation. In addition, comparative studies between accredited and non-accredited healthcare facilities, employing standardised performance metrics and adjusting for variables such as hospital size, case complexity, and resource availability, are essential to establish causal relations and quantify the magnitude of observed improvements.

Furthermore, qualitative investigations could provide valuable insights into the mechanisms by which accreditation influences clinical governance, organisational behaviour, and the engagement of healthcare personnel in continuous quality improvement (CQI) initiatives. Such studies may elucidate and role of accreditation in shaping institutional culture, enhancing interprofessional collaboration, and promoting adherence to evidence-based practices. A deeper understanding of these dynamics can inform refinement of CBAHI standards and support the development of targeted interventions to maximise their impact on patient safety and care quality within the Saudi healthcare system.

In parallel, comprehensive economic evaluations are warranted to examine the financial implications of implementing and maintaining CBAHI accreditation standards. These analyses should encompass both direct costs, such as infrastructure enhancements, staff training programs, and investment in health information systems and indirect costs, including opportunity costs and resource reallocation. Longitudinal cost assessment should be stratified by facility type, ownership (public vs. private), and size to capture variability in financial burden across institutions. Equally important is the evaluation of the return on investment (ROI) associated with accreditation, focusing on its potential to enhance operational efficiency, reduce preventable adverse events, shorten hospitalisation durations, and improve clinical outcomes. Additionally, accreditation may confer reputational benefit analysis that influences patient trust, satisfaction, and service utilisation rates. A balanced cost-benefit analysis that integrates both quantitative and qualitative data will be critical for informing strategic decision-making by hospital administration and policymakers. Such evidence can guide the sustainable implementation of accreditation programs and ensure that their adoption yields meaningful improvements in healthcare quality and safety throughout the KSA.

## Conclusions

This narrative review synthesised evidence on the implementation and outcomes of CBAHI accreditation across Saudi healthcare settings. Generally, accreditation was associated with meaningful improvements in infection control practices and selected patient safety outcomes, particularly in medication safety, incident reporting, and hand hygiene adherence. Most cross-sectional and audit studies were at moderate risk of bias due to sampling and self-report, while the interrupted time series offered stronger temporal inference but remained observational. Key enablers of successful implementation included strong leadership, staff training, and resource availability, while compliance barriers were associated with weak IT infrastructure, workforce shortages, and insufficient management support. However, gains in clinical outcomes and organisational culture were inconsistent, suggesting that accreditation alone is not sufficient to drive systemic transformation. To unlock its full potential, CBAHI must be embedded within continuous quality improvement frameworks, supported by digital infrastructure investment, leadership accountability, and sustained workforce development. Certainty is limited by small samples, cross-sectional designs, and heterogeneous outcome measures; future studies should use larger, multi-centre, longitudinal designs. Further research should focus on laborious, longitudinal evaluations and economic analyses to clarify the causal impact and cost-effectiveness of accreditation, thereby guiding strategic policy and institutional reform across the Kingdom’s evolving healthcare infrastructure. 
